# Inter-vendor reproducibility of left and right ventricular cardiovascular magnetic resonance myocardial feature-tracking

**DOI:** 10.1371/journal.pone.0193746

**Published:** 2018-03-14

**Authors:** Roman Johannes Gertz, Torben Lange, Johannes Tammo Kowallick, Sören Jan Backhaus, Michael Steinmetz, Wieland Staab, Shelby Kutty, Gerd Hasenfuß, Joachim Lotz, Andreas Schuster

**Affiliations:** 1 Department of Cardiology and Pneumology, Georg-August-University Göttingen, Göttingen, Germany; 2 DZHK (German Centre for Cardiovascular Research), partner site Göttingen, Göttingen, Germany; 3 Institute for Diagnostic and Interventional Radiology, Georg-August-University Göttingen, Göttingen, Germany; 4 Department of Paediatric Cardiology and Intensive Care Medicine, Georg-August-University Göttingen, Göttingen, Germany; 5 Children's Hospital and Medical Center, University of Nebraska, Omaha, NE, United States of America; 6 Department of Cardiology, Royal North Shore Hospital, The Kolling Institute, Northern Clinical School, University of Sydney, Sydney, Australia; Scuola Superiore Sant'Anna, ITALY

## Abstract

**Aim:**

Since cardiovascular magnetic resonance feature-tracking (CMR-FT) has been demonstrated to be of incremental clinical merit we investigated the interchangeability of global left and right ventricular strain parameters between different CMR-FT software solutions.

**Material and methods:**

CMR-cine images of 10 patients without significant reduction in LVEF and RVEF and 10 patients with a significantly impaired systolic function were analyzed using two different types of FT-software (TomTec, Germany; QStrain, Netherlands). Global longitudinal strains (LV GLS, RV GLS), global left ventricular circumferential (GCS) and radial strains (GRS) were assessed. Differences in intra- and inter-observer variability within and between software types based on single and up to three repeated and subsequently averaged measurements were evaluated.

**Results:**

Inter-vendor agreement was highest for GCS followed by LV GLS. GRS and RV GLS showed lower inter-vendor agreement. Variability was consistently higher in healthy volunteers as compared to the patient group. Intra-vendor reproducibility was excellent for GCS, LV GLS and RV GLS, but lower for GRS. The impact of repeated measurements was most pronounced for GRS and RV GLS on an intra-vendor level.

**Conclusion:**

Cardiac pathology has no influence on CMR-FT reproducibility. LV GLS and GCS qualify as the most robust parameters within and between individual software types. Since both parameters can be interchangeably assessed with different software solutions they may enter the clinical arena for optimized diagnostic and prognostic evaluation of cardiovascular morbidity and mortality in various pathologies.

## Introduction

Cardiovascular magnetic resonance feature tracking (CMR-FT) is a technique analogous to speckle tracking echocardiography (STE), a non-Doppler based technique to assess cardiac mechanics [1]. Quantitative wall motion parameters coming from STE demonstrate high value for prognosis and mortality prediction [2] over and above classical parameters such as ejection fraction (EF) [3]. Studies show good agreement between STE and CMR-FT [4–6] and recently similar utility for prognosis assessment has been demonstrated for CMR-FT [7–9]. Furthermore, reasonable agreement between CMR-FT and CMR-tagging [10], which is considered the CMR reference standard for quantitative wall motion assessment [11] has been demonstrated. Parameters derived from CMR-FT in contrast to CMR-tagging do not require the acquisition of additional sequences and time consuming post processing, but allow quantitative deformation parameters to be derived from routinely acquired steady-state-free-precession (SSFP) sequences [12]. CMR-FT has proven reliability [13, 14] and is receiving increasing interest due to mounting evidence regarding the clinical applicability in a variety of cardiovascular diseases [7, 8, 12, 15–20].

Notwithstanding these considerations significant numerical differences in strain assessments have been demonstrated between different CMR-FT software types (2D CPA MR, TomTec GmbH, Unterschleissheim, Germany and Tissue Tracking, cvi^42^, Circle Cardiovascular Imaging Inc., Calgary, Canada) [21]. Recently Medis Medical Imaging Systems (Leiden, Netherlands) have released an alternative tool called QStrain. Although both solutions share a similar basic algorithm [11], they offer different workflows with Medis requiring a higher degree of manual user interaction than TomTec. Consequently, the aim of the present study was to assess the reproducibility and inter-vendor agreement between the established TomTec methodology and the new solution provided by Medis in regard to global left and right ventricular strain values.

## Materials and methods

### Study population

The study cohort consisted of 10 patients with normal left ventricular ejection fraction (LVEF) and 10 patients with significantly impaired systolic function. The research was conducted in accordance with general ethical approval for additional research analyses on clinically acquired data granted by the Ethics committee of the University Medical Centre Goettingen. All patients gave written informed consent and all clinical investigations have been conducted according to the principles expressed in the Declaration of Helsinki.

### CMR imaging

CMR imaging was carried out on a SIEMENS Symphony 1.5 Tesla system in the supine position using a five-channel cardiac surface coil. Electrocardiogram (ECG)-gated SSFP cine sequences in long-axis 2- and 4-chamber views and 12 to 14 equidistant short-axis planes completely covering the left ventricle were acquired during brief periods of breath-holding (25 frames/cardiac cycle). Typical CMR parameters were as follows: pixel spacing: 1.6mm x 1.6mm; 7 mm slice thickness; 8 mm inter-slice distance; TE: 1.4ms; TR: 46ms.

### Volumetric analysis

CMR based volumetric analysis was performed using the dedicated software solution provided by Medis Medical Imaging Systems (QMass, Version 7.6).

### CMR-Feature Tracking (CMR-FT)

CMR-FT was performed using the software provided by TomTec Imaging Systems (2D CPA MR, Cardiac Performance Analysis, Version 4.6.3.9) and Medis Medical Imaging Systems (QStrain, Version 2.1.12.2). The software tools will be referred to as “TomTec” and “QStrain” in the following sections of the paper. Strain was assessed with both software types at the following locations: long axis 2- and 4-chamber views; short axis sections at basal, mid-ventricular and apical levels. The slices of the different short axis levels were identified as follows; basal level: last slice showing the complete left ventricular myocardium throughout the entire cardiac circle without in plane appearance of the left ventricular outflow tract (LVOT) at end-systole; mid-ventricular: slice located at the level of both papillary muscles; apical: slice showing consisting blood-pool cavity throughout the entire cardiac cycle (no obliteration of the lumen at end-systole). RV tracking was performed including the septum.

With TomTec left ventricular (LV) endocardial and epicardial borders were manually delineated at short and long-axis views with the initial contour set at end-diastole. Due to the thin myocardial wall right ventricular (RV) tracking was performed only delineating an endocardial contour. Workflow using QStrain was different since the software introduces the work step to delineate cardiac contours both at end-diastole and end-systole. In case of insufficient tracking, as defined by apparent deviations of the contours from the endocardial and/or epicardial borders, contours were manually corrected and the algorithm reapplied. All measurements were repeated three times in all sections.

All patients were analyzed by one single observer (RJG) using both types of software. The same observer repeated the analysis on the same data-sets four weeks later to assess intra-observer variability. Inter-observer reproducibility was derived from the tracking results of a second skilled observer (TL). To study the impact of repeated measurements on reproducibility results based on a single measurement (R1) were compared with the results for these parameters derived from two (R2) and three (R3) repeated and subsequently averaged measurements.

### Statistical analysis

Microsoft Excel and IBM SPSS Statistics version 23 for Mac were used to conduct statistical analysis. All continuous data are reported as mean ± standard deviation. Statistical parameters to assess inter-vendor agreement and intra- and inter-observer variability were calculated as follows: Bland-Altman analysis [22] (mean difference between measurements with 95% limits of agreement (±1.96 standard deviations)), intra-class correlation coefficients (ICC) using a model of absolute agreement (agreement was considered excellent when ICC > 0.74, good when ICC  =  0.60–0.74, fair when ICC  =  0.40–0.59, and poor when ICC < 0.4 [23]) and the coefficient of variation (CoV) (defined as the standard deviation of the differences divided by the mean [24]). The Kolmogorov-Smirnov test was applied to test for normal distribution of the data [25]. To compare mass and volumetric parameters between healthy volunteers and the patient group parametric parameters were tested according to the *t*-test, while the Man-Whitney *U* test was applied for non-parametric data. Pairwise non-parametric strain parameters assessed with each vendor were compared using the Wilcoxon test. The Mann-Whitney *U* test was used to analyze whether there was a significant difference between non-parametric strain parameters for healthy volunteers and patients with impaired cardiac function, respectively. Significance was defined as p < 0.05.

## Results

### Participant details

**Fig 1:**
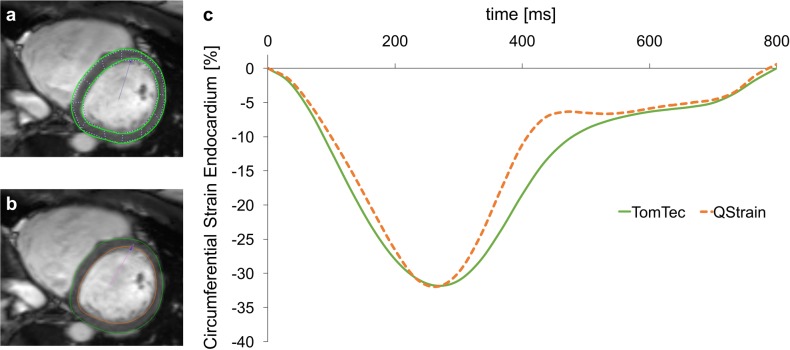
Example of LV circumferential strain assessments at the basal level and corresponding strain curves. Example of LV circumferential strain assessments at the basal level with TomTec (*Panel a*) and QStrain (*Panel b*). Manually contoured epicardial and endocardial borders and respective strain curves (*Panel c*) are being displayed for both types of software.

Demographics are displayed in Table 1. Quantitative analyses were performed in all subjects, no subject was excluded. Fig 1 shows representative assessments of LV circumferential strains at basal level with both software types including contours and corresponding strain curves.

**Table 1:** Patient demographics.

Demographics		Normal subjects	Patients with reduced cardiac function*	*P* value
Study population, *n*		10	10	
Gender (F/M)		6/4	6/4	1.00
Age (years)		37 (20–62)	47 (21–80)	0.14
LVEF (%)		69.0 (3.3)	37.0 (9.8)	<0.01
LV mass index (g/m^2^)		49.09 (14.78)	68.20 (28.32)	0.01
LV EDVI (ml/m^2^)		74.79 (13.03)	122.15 (28.95)	<0.01
LV ESVI (ml/m^2^)		23.21 (5.63)	78.29 (29.20)	<0.01
RVEF (%)		59.4 (7.1)	40.0 (1.3)	<0.01
RV mass index (g/m^2^)		12.01 (5.21)	14.42 (5.37)	0.10
RV EDVI (ml/m^2^)		71.35 (16.00)	96.29 (38.20)	0.23
RV ESVI (ml/m^2^)		29.09 (10.84)	59.78 (31.05)	0.02
Means LV GLS %	TomTec	-22.57 (5.13)	-11.54 (3.18)	<0.01
	QStrain	-23.98 (2.90)	-12.14 (3.55)	<0.01
Mean GCS %	TomTec	-31.44 (4.05)	-15.30 (4.48)	<0.01
	QStrain	-33.01 (3.14)	-15.04 (5.27)	<0.01
Mean GRS %	TomTec	24.67 (5.21)	13.78 (4.90)	<0.01
	QStrain	41.33 (8.54)	21.43 (11.28)	<0.01
Mean RV GLS %	TomTec	-23.74 (6.05)	-11.75 (4.25)	<0.01
	QStrain	-26.16 (6.18)	-14.88 (4.99)	<0.01

Continuous variables are expressed as mean (standard deviation). Age is expressed as median (range). Volumetric results have been measured by MRI volumetry. Results for mean strain parameters are given based on three averaged measurements (R3). LVEF, left ventricular ejection fraction; LV EDVI, left ventricular enddiastolic volume; LV ESVI, left ventricular endsystolic volume; RVEF, right ventricular ejection fraction; RV EDVI, right ventricular enddiastolic volume; RV ESVI, right ventricular endsystolic volume; LV GLS, global left ventricular longitudinal strain; GCS, global left ventricular circumferential strain; GRS, global left ventricular radial strain; RV GLS, global right ventricular longitudinal strain

*as defined by reduced ejection fraction

### Inter-vendor agreement

**Fig 2:**
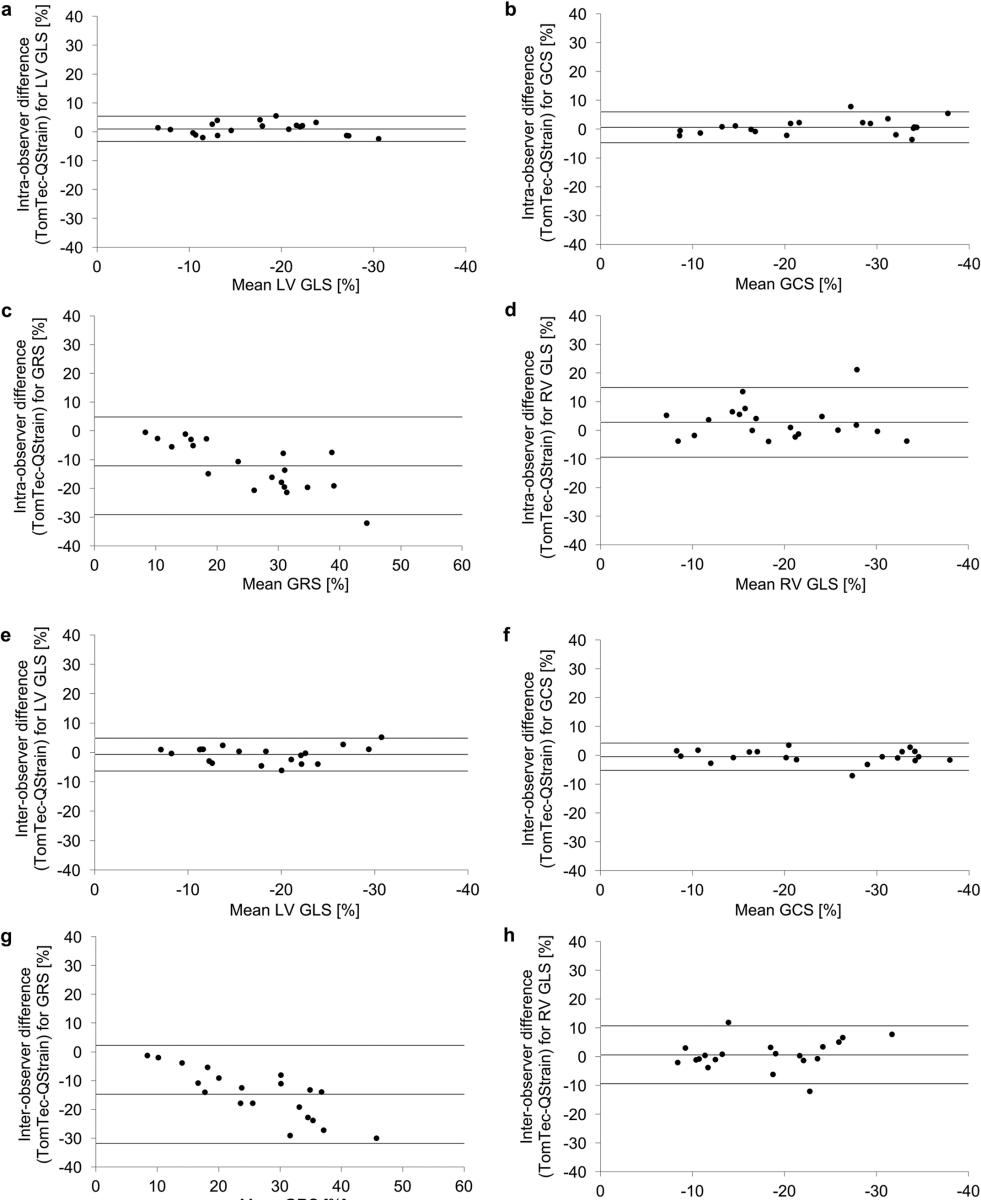
Reproducibility for CMR-FT derived global strain parameters at intra- and inter-observer levels. Inter-vendor agreement for global strain parameters for healthy volunteers and patients with impaired cardiac output based on three averaged measurements (R3). *Panel a–d*: Bland-Altman plots with limits of agreement (95% confidence intervals) demonstrating the CMR-FT derived reproducibility at an intra-observer level are being displayed. *Panel e–h*: Bland-Altman plots with limits of agreement (95% confidence intervals) demonstrating the CMR-FT derived reproducibility at an inter-observer level are being displayed.

Table 2 summarizes values for mean difference ± standard deviation, ICC and CoV for all strain-parameters based on three averaged measurements (R3). Corresponding Bland-Altman plots are displayed in Fig 2.

**Table 2:** Inter-vendor agreement and intra-vendor reproducibility at intra- and inter-observer levels for global longitudinal, global circumferential and global radial strain based on three averaged measurements (R3).

		TomTec versus QStrain			TomTec			QStrain		
		Mean Difference (SD of the Diff.)	ICC (95% CI)	CoV (%)	Mean Difference (SD of the Diff.)	ICC (95% CI)	CoV (%)	Mean Difference (SD of the Diff.)	ICC (95% CI)	CoV (%)
Intra-observer	LV GLS %	1.00 (2.23)	0.97 (0.92–0.99)	12.70	-0.15 (0.64)	1.00 (1.00–1.00)	3.79	0.24 (0.87)	1.00 (0.99–1.00)	4.78
	GCS %	0.66 (2.73)	0.98 (0.95–1.00)	11.50	-0.49 (0.56)	1.00 (0.99–1.00)	2.41	0.03 (0.74)	1.00 (1.00–1.00)	3.08
	GRS %	-12.16 (8.67)	0.62 (0.00–0.88)	34.28	-0.85 (3.00)	0.96 (0.90–0.99)	15.29	-2.45 (4.59)	0.97 (0.90–0.99)	14.07
	RV GLS %	2.78 (6.21)	0.80 (0.49–0.92)	32.47	-0.62 (1.14)	0.99 (0.98–1.00)	6.55	0.59 (1.38)	0.99 (0.98–1.00)	6.61
Inter-observer	LV GLS %	-0.72 (2.88)	0.96 (0.89–0.98)	16.03	0.52 (0.94)	1.00 (0.98–1.00)	5.41	0.18 (0.72)	1.00 (0.99–1.00)	3.96
	GCS %	-0.51 (2.40)	0.99 (0.96–0.99)	10.08	0.17 (1.09)	1.00 (0.99–1.00)	4.64	0.23 (0.91)	1.00 (1.00–1.00)	3.76
	GRS %	-14.78 (8.72)	0.53 (0.00–0.85)	32.98	0.17 (3.00)	0.96 (0.89–0.98)	15.67	4.51 (6.87)	0.89 (0.64–0.96)	23.59
	RV GLS %	0.63 (5.12)	0.86 (0.65–0.95)	28.75	0.40 (1.13)	0.99 (0.99–1.00)	6.30	0.19 (0.80)	1.00 (0.99–1.00)	3.89

SD, standard deviation; Diff., differences; ICC, intra-class correlation coefficient; CoV, coefficient of variation; CI, confidence interval; LV GLS, global left ventricular longitudinal strain; GCS, global left ventricular cirucumferential strain; GRS, global left ventricular radial strain; RV GLS, global right ventricular longitudinal strain

Inter-vendor agreement was excellent for GCS and LV GLS for both intra- and inter-observer levels. RV GLS and GRS both showed lower inter-vendor agreement.

There was no significant difference between vendors regarding the averaged results (R3) for LV GLS (p = 0.079), GCS (p = 0.502) and RV GLS (p = 0.093). GRS measured with QStrain was significantly higher (p < 0.001) than measured with TomTec. These findings were similar for the analysis based on a single repetition (R1) and two averaged repetitions (R2) for LV GLS, GCS and GRS. RV GLS based on a single measurement (R1) only, however, was significantly lower measured with TomTec (p = 0.033).

### Reproducibility

All values for mean difference ± standard deviation, ICC and CoV as derived from three averaged measurements (R3) are given in Table 2. In both vendors, intra-vendor reproducibility was best for GCS followed by LV GLS. RV GLS showed excellent intra-vendor reproducibility with both types of software. GRS showed the highest intra-vendor variability amongst all parameters. Whilst TomTec showed higher intra- than inter-observer reproducibility, inter-observer reproducibility with QStrain was slightly better than intra-observer reproducibility.

### Impact of repeated measurements on reproducibility

**Fig 3:**
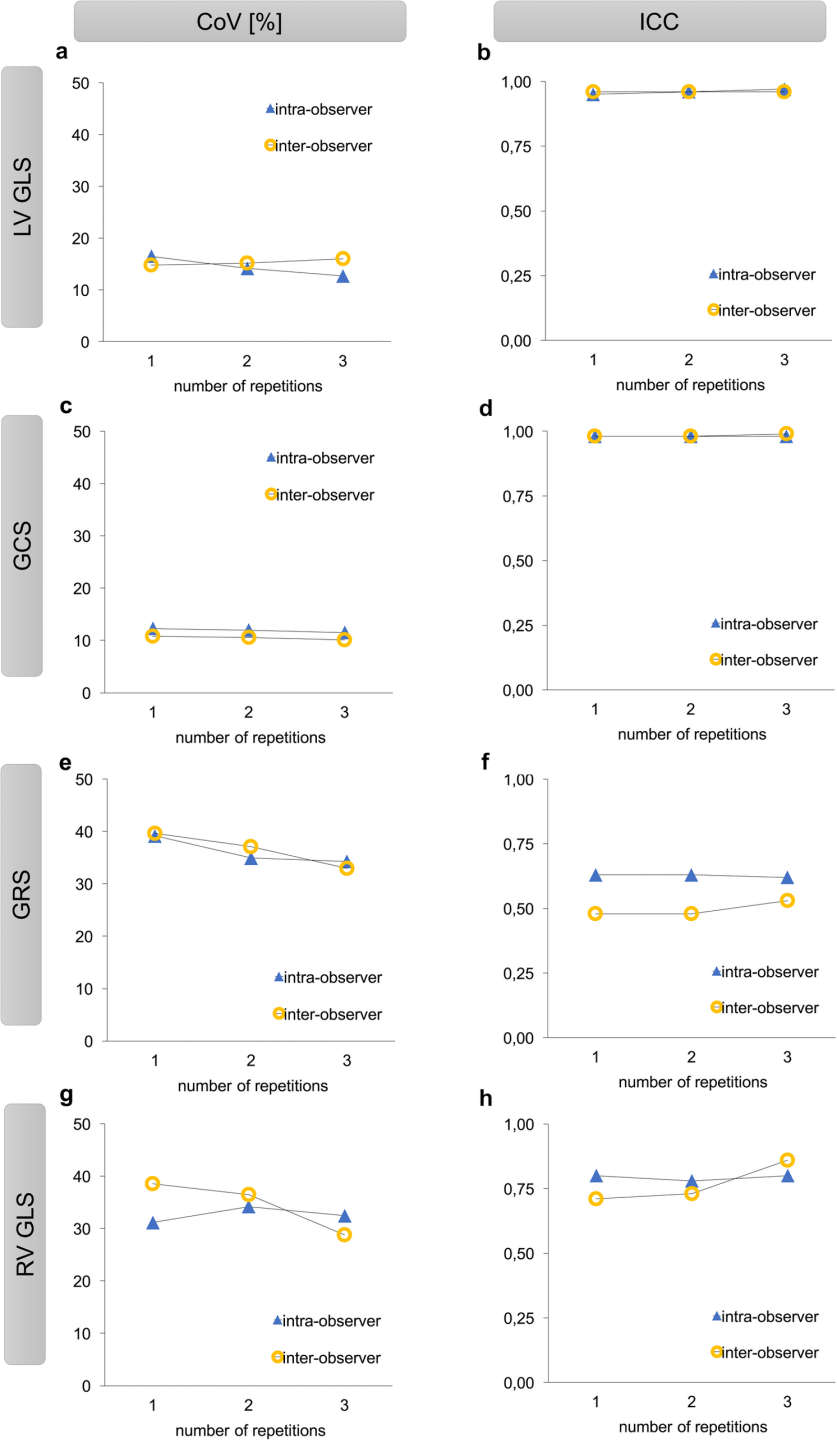
Impact of repeated measurements on reproducibility. *Panel a–h*. Inter-vendor agreement based on CoV [%] and ICC for one, two and three averaged measurements. CoV [%], coefficient of variation; ICC, intra-class correlation coefficient; LV GLS, global left ventricular longitudinal strain; GCS, global left ventricular circumferential strain; GRS, global left ventricular radial strain; RV GLS, global right ventricular longitudinal strain.

Table 3 displays the results for inter-vendor agreement and intra-observer variability based on one measurement (R1) as compared to two averaged measurements (R2, Table 4) and three averaged measurements (R3, Table 2). Fig 3 shows the impact of repeated measurements on inter-vendor agreement based on CoV and ICC. Repeated measurements had moderate impact on LV GLS and GCS regarding inter-vendor agreement and intra-vendor variability (Tables 2, 3 and 4). The effect was more pronounced for RV GLS and GRS regarding intra-vendor reproducibility but not inter-vendor agreement (Tables 2, 3 and 4).

**Table 3:** Inter-vendor agreement and intra-vendor reproducibility at intra- and inter-observer levels for global longitudinal, global circumferential and global radial strain based on one measurement (R1).

		TomTec versus QStrain			TomTec			QStrain		
		Mean Difference (SD of the Diff.)	ICC (95% CI)	CoV (%)	Mean Difference (SD of the Diff.)	ICC (95% CI)	CoV (%)	Mean Difference (SD of the Diff.)	ICC (95% CI)	CoV (%)
Intra-observer	LV GLS %	1.06 (2.92)	0.95 (0.88–0.98)	16.49	-0.09 (1.02)	1.00 (0.99–1.00)	5.97	0.16 (1.10)	1.00 (0.99–1.00)	6.00
	GCS %	0.82 (2.91)	0.98 (0.94–0.99)	12.29	-0.55 (0.82)	1.00 (0.99–1.00)	3.57	0.09 (1.00)	1.00 (0.99–1.00)	4.13
	GRS %	-12.57 (10.07)	0.63 (0.00–0.88)	39.16	-0.36 (4.42)	0.93 (0.83–0.97)	22.52	-2.68 (8.22)	0.92 (0.80–0.97)	24.64
	RV GLS %	3.17 (5.98)	0.80 (0.47–0.92)	31.15	-0.35 (1.64)	0.99 (0.97–1.00)	9.39	0.59 (1.53)	0.99 (0.98–1.00)	7.25
Inter-observer	LV GLS %	0.59 (2.68)	0.96 (0.91–0.99)	14.80	0.64 (1.35)	0.99 (0.97–1.00)	7.75	-0.08 (1.14)	0.99 (0.98–1.00)	6.25
	GCS %	0.92 (2.56)	0.98 (0.95–0.99)	10.80	0.00 (1.11)	1.00 (0.99–1.00)	4.75	-0.07 (1.01)	1.00 (0.99–1.00)	4.21
	GRS %	-15.55 (10.66)	0.48 (0.00–0.81)	39.62	0.29 (3.37)	0.95 (0.87–0.98)	17.49	4.85 (8.58)	0.87 (0.62–0.95)	29.00
	RV GLS %	2.92 (7.68)	0.71 (0.29–0.88)	38.57	0.84 (2.46)	0.97 (0.93–0.99)	13.61	0.39 (1.56)	0.99 (0.98–1.00)	7.42

SD, standard deviation; Diff., differences; ICC, intra-class correlation coefficient; CoV, coefficient of variation; CI, confidence interval; LV GLS, global left ventricular longitudinal strain; GCS, global left ventricular circumferential strain; GRS, global left ventricular radial strain; RV GLS, global right ventricular longitudinal strain

**Table 4:** Inter-vendor agreement and intra-vendor reproducibility at intra- and inter-observer levels for global longitudinal, global circumferential and global radial strain based on two averaged measurements (R2).

		TomTec versus QStrain			TomTec			QStrain		
		Mean Difference (SD of the Diff.)	ICC (95% CI)	CoV (%)	Mean Difference (SD of the Diff.) (SD of the Diff.)	ICC (95% CI)	CoV (%)	Mean Difference (SD of the Diff.) (SD of the Diff.)	ICC (95% CI)	CoV (%)
Intra-observer	LV GLS %	0.96 (2.49)	0.96 (0.91–0.99)	14.14	-0.04 (0.74)	1.00 (0.99–1.00)	4.30	0.27 (0.87)	1.00 (0.99–1.00)	4.77
	GCS %	0.77 (2.84)	0.98 (0.95–0.99)	11.96	-0.53 (0.65)	1.00 (0.99–1.00)	2.82	-0.01 (0.77)	1.00 (1.00–1.00)	3.20
	GRS %	-12.51 (8.93)	0.63 (0.00–0.88)	34.91	-0.69 (2.98)	0.96 (0.90–0.99)	15.15	-2.91 (5.36)	0.96 (0.87–0.99)	16.10
	RV GLS %	2.71 (6.53)	0.78 (0.46–0.91)	34.18	-0.58 (1.31)	0.99 (0.98–1.00)	7.48	0.74 (1.36)	0.99 (0.97–1.00)	6.52
Inter-observer	LV GLS %	0.76 (2.73)	0.96 (0.90–0.98)	15.17	0.47 (0.91)	1.00 (0.99–1.00)	5.72	0.01 (1.03)	1.00 (0.99–1.00)	5.66
	GCS %	0.91 (2.52)	0.98 (0.95–0.99)	10.59	-0.06 (0.88)	1.00 (1.00–1.00)	3.76	0.15 (0.96)	1.00 (1.00–1.00)	3.95
	GRS %	-15.70 (9.98)	0.48 (0.00–0.82)	37.13	0.28 (2.77)	0.96 (0.90–0.98)	14.44	4.65 (7.81)	0.88 (0.63–0.96)	26.47
	RV GLS %	3.11 (7.24)	0.73 (0.34–0.89)	36.49	0.52 (1.24)	0.99 (0.98–1.00)	6.88	0.26 (1.02)	1.00 (0.99–1.00)	4.94

SD, standard deviation; Diff., differences; ICC, intra-class correlation coefficient; CoV, coefficient of variation; CI, confidence interval; LV GLS, global left ventricular longitudinal strain; GCS, global left ventricular circumferential strain; GRS, global left ventricular radial strain; RV GLS, global right ventricular longitudinal strain

### Inter-vendor agreement in health and disease

Table 5 reports inter-vendor agreement based on mean differences with limits of agreement for the whole study cohort, healthy volunteers and patients with impaired cardiac function. Corresponding Bland-Altman Plots are displayed in Fig 2 and in the Supporting information in S1 Fig and S2 Fig. Inter-vendor agreement was sufficient in patients and healthy volunteers (Supporting information S1 Fig and S2 Fig). Inter-vendor agreement was higher for all global strain parameters in the patient group as compared to the volunteers (Table 5). This applied to the results on an intra-observer and on an inter-observer level. Consistently, LV GLS and GCS were found to show the best inter-vendor agreement in patients and healthy volunteers, respectively. In all groups agreement between vendors was reasonable for RV GLS and lower for GRS. This was paralleled by similar results on intra-vendor levels both for TomTec and for QStrain (see Table 2, S1 and S2 Tables).

**Table 5:** Inter-vendor agreement on an intra- and inter-observer level for global longitudinal, global circumferential andglobal radial strain for the complete study cohort, normal subjects and patients with impaired cardiac function* based on three averaged measurements (R3).

		complete study cohort	normal subjects	patients with impaired
				cardiac function*
		Mean Difference (LOA)	Mean Difference (LOA)	Mean Difference (LOA)
Intra-observer	LV GLS %	1.00 (-3.37–5.37)	1.40 (-3.61–6.42)	0.60 (-3.12–4.31)
	GCS %	0.66 (-4.69–6.00)	1.57 (-5.01–8.14)	-0.26 (-3.41–2.90)
	GRS %	-12.16 (-29.16–4.84)	-16.66 (-31.01 –-2.32)	-7.65 (-22.85 –-7.65)
	RV GLS %	2.78 (-9.40–14.95)	2.42 (-11.42–16.27)	3.13 (-7.83–14.09)
Inter-observer	LV GLS %	-0.72 (-6.36–4.91)	-1.40 (-8.41–5.61)	-0.05 (-3.78–3.69)
	GCS %	-0.51 (-5.21–4.19)	-1.18 (-6.60–4.25)	0.15 (-3.52–3.82)
	GRS %	-14.78 (-31.88–2.31)	-19.84 (-35.37 –-4.31)	-9.73 (-22.28–2.82)
	RV GLS %	0.63 (-9.42–10.67)	0.51 (-11.39–12.41)	0.74 (-7.70–9.18)

LOA, limits of agreement; LV GLS, global left ventricular longitudinal strain; GCS, global left ventricular circumferential strain; GRS, global left ventricular radial strain; RV GLS, global right ventricular longitudinal strain

*as defined by reduced ejection fraction

## Discussion

To our knowledge this is the first study investigating the performance and potential differences between the recently introduced CMR-FT tool QStrain and the more established TomTec software, that has already been used and validated in a variety of studies [6, 21, 26].

First, our findings show reasonable inter-vendor agreement between both types of software. GCS and LV GLS qualify as the best parameters with excellent reproducibility and interchangeability based on a single analysis. Second, RV GLS and GRS are less robust with significant inter-vendor variability. However, intra-vendor reproducibility of these parameters can be improved by repeated analysis runs resulting in sufficient reproducibility when using a single software type. Consequently, it is important staying within one vendor when calculating these parameters. Third, we could show that reproducibility of CMR-FT within and between software types is not adversely affected by impaired ventricular function.

Our results consistently indicate the high clinical applicability of GCS and LV GLS as most robust parameters in both types of software, which is in line with previously published literature [12–14, 16]. Similar to the current study, GCS has been repeatedly shown to have the least variability in previous CMR-FT studies [13], earlier studies comparing STE and CMR-FT [4, 5] and tagging and CMR-FT [27]. Buss et al. found CMR-FT derived LV GLS and GCS to serve as a predictors of cardiac events, independent of clinical and laboratory markers, LVEF and late gadolinium enhancement in patients with dilated cardiomyopathy [7]. More recently, Orwat et al. could show that in patients with repaired tetralogy of Fallot LV GLS and GCS are significantly associated with outcome [8]. This growing evidence for LV GLS and GCS as prognostic tools in a variety of diseases and their high interchangeability between different vendors based on a single analysis underline their potential prospective incremental clinical merit.

RV GLS and GRS have already been reported in previous studies to show lower inter-vendor reproducibility than GCS and LV GLS [21]. This was in line with our findings. GRS represents strain throughout the entire myocardial wall from subepicardium to subendocardium and is consequently much more affected by through plane motion [21] and complex diastolic and systolic twisting motion [28] than LV GLS and GCS that are predominantly assessed with subendocardial tracking. The two software solutions, which were applied in the present study rely on a similar algorithm, which explains the good agreement for LV GLS, GCS and RV GLS. A higher degree of manual user interaction (e.g. manual defining of both, systolic and diastolic contours) as required by QStrain did not impact reproducibility. As mentioned above the lower reproducibility for GRS, however is inherent to FT algorithms based on optical flow methods as previously shown [13, 14, 21, 27]. Interestingly a recently introduced CMR-FT software (Segment CMR by Medviso, Lund, Sweden) using an algorithm which incorporates non-rigid image registration [29, 30] seems to overcome this limitation as suggested by Morais et. al. [31]. Instead of tracking endocardial borders only this new algorithm tracks the entire image content (i.e. blood pool and the entire myocardium) and thus a higher number of myocardial image samples. As mentioned before GRS represents strain throughout the entire myocardial wall and is therefore the strain parameter that is particularly affected by this different approach. Morais et al. could show that this leads to a significantly better reproducibility for GRS than reported in all previous studies that were carried out with CMR-FT software which did not incorporate non-rigid image registration [7, 13, 14, 16, 21, 27].

Notwithstanding these considerations, intra-vendor agreement for RV GLS can be improved through repeated runs and was excellent in both types of software based on three averaged measurements. Thus, our study points out two important aspects: first, both software solutions can serve as a reliable tool in the assessment of CMR-FT derived right ventricular strain; second, three averaged runs make results for RV GLS significantly more reliable and might justify a threefold increased analysis time in future studies when assessing right ventricular strain. This is an important finding since the role of strain deterioration in right ventricular pathologies is of growing research interest with potential clinical utility. For instance recently published studies indicate that CMR-FT derived RV GLS analyses play a promising role in the detection of right ventricular pathologies such as arrhythmogenic right ventricular cardiomyopathy (ARVC) [17] and even allow a prediction of subsequent clinical deterioration in diseases affecting the right ventricle such as pulmonary hypertension [19]. In addition, RV GLS was recently identified as a predictor of outcome in patients with repaired tetralogy of Fallot [8].

Our data indicate no significant differences between TomTec and QStrain comparing values for LV GLS, GCS and RV GLS. Earlier vendor-comparisons using TomTec and Circle, cvi^42^ (Circle) found interchangeability for GCS between these vendors to be limited, because values for GCS measured with Circle were significantly lower than assessed with TomTec [21]. As a result, interchangeability between TomTec and QStrain is superior to the interchangeability between TomTec and Circle. However, it is important to note that regardless of the number of repetitions applied GRS results are not interchangeable as values for this parameter were significantly higher measured with QStrain. This difference between vendors was, on the contrary, not found comparing TomTec and Circle [21]. Taking into account normal values for GRS according to a review by Claus et. al [32]) and the range for GRS observed in this study cohort (8% to 61%), a mean difference between vendors of 12.16% (Table 2) appears quite considerable. Thus, serial GRS assessments with both software types e.g. during patient follow-up at different hospitals do not seem to represent a valid clinical methodology. Since the intra-vendor reproducibility is adequate one needs to decide which software to use consistently when analyzing GRS in future studies to avoid poor interchangeably between vendors and to allow GRS to be quantified with accurate performance.

In line with the findings from STE studies [33] reproducibility was even higher in patients with heart failure than in healthy volunteers. These findings appear intuitive as strain in healthy volunteers is usually higher than in patients with impaired cardiac function, which was also the case in the current study (Table 1). Higher strain parameters indicate more cardiac motion and are thus much stronger affected by through plane motion effects, which are known to lower reproducibility at the regional level [13, 34, 35]. However, contradictory to our results the study by Morais et al. has shown that intra-observer, inter-observer and inter-study variability is similar in healthy volunteers and patients with known myocardial pathology [31]. When interpreting these differences, it is important to note that first the technique used by Morais et al. is based on a different CMR-FT algorithm and second LVEF in the patient group analyzed by Morais et al. was significantly higher than in our patient group. Moreover, to divide study groups based on LVEF might represent a confounding factor for the comparison of reproducibility in health and disease as there is evidence from echocardiographic studies [36, 37] and other CMR-FT studies [17] that strain parameters can be impaired, when global function parameters may still be normal.

When interpreting the results of the current study, it is important to bear in mind that the main step for the assessment of myocardial strain with CMR-FT is the initial and manual delineation of the endo- and epicardial contour by a skilled observer. Although the identification of these contours can be performed easily and quickly some variation between two different observers is inherent to the process and thus a potential source of variability. Moreover, the process is complicated by the fact that rotational and strain metrics estimates neglect out-of-plane movement of the myocardium throughout the heart cycle when using 2 dimensional techniques. Ideally, further refinements should aim at the development of 3 dimensional techniques with fully automatic analysis solutions to overcome these limitations.

### Study limitations

Even though our study showed reasonable inter-vendor agreement for global strain parameters the results have to be interpreted with caution. First and foremost, the study population was quite small and patients and healthy volunteers were divided into each group only regarding to their LVEF, while right ventricular performance or etiology for an impairment of the LVEF was of no concern. Furthermore, both groups were not age matched, however, the age differences between both groups according to the *t*-test were not significant (p = 0.14). Besides distribution between sexes was balanced between the two groups but not within each group. However, the study did not aim at any quantitative comparison of strain parameters between health and disease, nor according to the etiology for the impairment of cardiac function, age or sex. Additionally, no true blinding of the observers as to whether a subject belonged to the healthy volunteer or the patient group could be achieved since a marked reduction in systolic function can be easily appreciated from the original images. However, all observers were blinded as well to any of their own results as to the results of the second observer. Off note, in the present study GCS and GRS were derived from the exactly same slices by all observers. It is important to note that especially for the mid-ventricular and the apical slices there might be a second or even a third slice that would have met the specified slice selection criteria. Thus a different slice selection among different observers is a possible source of variability in clinical practice that is not reflected in the results of the current study. Notwithstanding this consideration this study aimed to quantify the variability inherent to the tracking performance rather than the variability that is introduced by slice selection.

As no echocardiographic or CMR-tagging was performed in any of the patients the study did not include any independent reference standard. Notwithstanding, TomTec has been validated against myocardial tagging with excellent agreement [10] and speckle tracking echocardiography with reasonable to good agreements in earlier studies [4–6]. Besides, we did not aim at another comparison between different techniques to assess cardiac dynamics but rather at an inter-vendor comparison between the two types of commercially available CMR-FT software to clarify how well they agree with each other and to what degree results can be used interchangeably.

## Conclusion

In conclusion, our study shows reasonable inter-vendor agreement between both types of software without negative affection of reproducibility when studying either healthy subjects or patients with cardiac pathology. LV GLS and GCS qualify as the most robust parameters and can be used interchangeably based on single measurements only. When analyzing right ventricular strain with either vendor three repeated runs are highly recommendable to improve reproducibility. Independent of the number of repetitions interchangeability of RV GLS and GRS may be questioned based on our results. Consequently, one should stay within one vendor when assessing these parameters.

If further studies will be able to confirm these findings CMR-FT derived quantitative deformation parameters may be fully implemented within routine clinical MR examinations for optimized diagnostic assessments and risk prediction in various cardiac pathologies.

## Supporting information

S1 Fig Reproducibility for CMR-FT derived global strain parameters at intra- and inter-observer levels for normal subjects. Inter-vendor agreement for global strain parameters for normal subjects based on three averaged measurements (R3). *Panel a–d*: Bland-Altman plots with limits of agreement (95% confidence intervals) demonstrating the CMR-FT derived reproducibility at an intra-observer level are being displayed. *Panel e–h*: Bland-Altman plots with limits of agreement (95% confidence intervals) demonstrating the CMR-FT derived reproducibility at an inter-observer level are being displayed.
(TIF)

S2 Fig Reproducibility for CMR-FT derived global strain parameters at intra- and inter-observer levels for patients with impaired cardiac function. Inter-vendor agreement for global strain parameters for patients with impaired cardiac function as defined by reduced ejection fraction based on three averaged measurements (R3). *Panel a–d*: Bland-Altman plots with limits of agreement (95% confidence intervals) demonstrating the CMR-FT derived reproducibility at an intra-observer level are being displayed. *Panel e–h*: Bland-Altman plots with limits of agreement (95% confidence intervals) demonstrating the CMR-FT derived reproducibility at an inter-observer level are being displayed.
(TIF)

S1 Table Inter-vendor agreement and intra-vendor reproducibility at intra- and inter-observer levels for global longitudinal, global circumferential and global radial strain for normal subjects based on three averaged measurements (R3). SD, standard deviation; Diff., differences; ICC, intra-class correlation coefficient; CoV, coefficient of variation; CI, confidence interval; LV GLS, global left ventricular longitudinal strain; GCS, global left ventricular circumferential strain; GRS, global left ventricular radial strain; RV GLS, global right ventricular longitudinal strain.
(DOCX)

S2 Table Inter-vendor agreement and intra-vendor reproducibility at intra- and inter-observer levels for global longitudinal, global circumferential and global radial strain for patients with impaired cardiac function* based on three averaged measurements (R3). SD, standard deviation; Diff., differences; ICC, intra-class correlation coefficient; CoV, coefficient of variation; CI, confidence interval; LV GLS, global left ventricular longitudinal strain; GCS, global left ventricular circumferential strain; GRS, global left ventricular radial strain; RV GLS, global right ventricular longitudinal strain; *as defined by reduced ejection fraction.
(DOCX)

## References

[1] Suffoletto MS, Dohi K, Cannesson M, Saba S, Gorcsan J. Novel Speckle-Tracking Radial Strain From Routine Black-and-White Echocardiographic Images to Quantify Dyssynchrony and Predict Response to Cardiac Resynchronization Therapy. Circulation. 2006;113(7):960–8. doi: 10.1161/CIRCULATIONAHA.105.571455 16476850 10.1161/CIRCULATIONAHA.105.571455

[2] Russo C, Jin Z, Elkind MS, Rundek T, Homma S, Sacco RL, et al Prevalence and prognostic value of subclinical left ventricular systolic dysfunction by global longitudinal strain in a community‐based cohort. European journal of heart failure. 2014;16(12):1301–9. doi: 10.1002/ejhf.154 25211239 10.1002/ejhf.154 PMC4672867

[3] Stanton T, Leano R, Marwick TH. Prediction of All-Cause Mortality From Global Longitudinal Speckle Strain: Comparison With Ejection Fraction and Wall Motion Scoring. Circulation: Cardiovascular Imaging. 2009;2(5):356–64. doi: 10.1161/CIRCIMAGING.109.862334 19808623 10.1161/CIRCIMAGING.109.862334

[4] Amaki M, Savino J, Ain DL, Sanz J, Pedrizzetti G, Kulkarni H, et al Diagnostic Concordance of Echocardiography and Cardiac Magnetic Resonance–Based Tissue Tracking for Differentiating Constrictive Pericarditis From Restrictive Cardiomyopathy. Circulation: Cardiovascular Imaging. 2014;7(5):819–27. 25107553 10.1161/CIRCIMAGING.114.002103

[5] Padiyath A, Gribben P, Abraham JR, Li L, Rangamani S, Schuster A, et al Echocardiography and Cardiac Magnetic Resonance‐Based Feature Tracking in the Assessment of Myocardial Mechanics in Tetralogy of Fallot: An Intermodality Comparison. Echocardiography. 2013;30(2):203–10. doi: 10.1111/echo.12016 23167248 10.1111/echo.12016

[6] Kempny A, Fernandez-Jimenez R, Orwat S, Schuler P, Bunck AC, Maintz D, et al Quantification of biventricular myocardial function using cardiac magnetic resonance feature tracking, endocardial border delineation and echocardiographic speckle tracking in patients with repaired tetralogy of Fallot and healthy controls. J Cardiovasc Magn Reson. 2012;14:32 doi: 10.1186/1532-429X-14-32 ; PubMed Central PMCID: PMCPMC3464868. 22650308 10.1186/1532-429X-14-32 PMC3464868

[7] Buss SJ, Breuninger K, Lehrke S, Voss A, Galuschky C, Lossnitzer D, et al Assessment of myocardial deformation with cardiac magnetic resonance strain imaging improves risk stratification in patients with dilated cardiomyopathy. European Heart Journal-Cardiovascular Imaging. 2014;16(3):307–15. doi: 10.1093/ehjci/jeu181 25246506 10.1093/ehjci/jeu181

[8] Orwat S, Diller G-P, Kempny A, Radke R, Peters B, Kühne T, et al Myocardial deformation parameters predict outcome in patients with repaired tetralogy of Fallot. Heart. 2016;102(3):209 doi: 10.1136/heartjnl-2015-308569 26715570 10.1136/heartjnl-2015-308569

[9] Eitel I, Stiermaier T, Lange T, Rommel K-P, Koschalka A, Kowallick JT, et al Cardiac Magnetic Resonance Myocardial Feature Tracking for Optimized Prediction of Cardiovascular Events Following Myocardial Infarction. JACC: Cardiovascular Imaging. 2018 doi: 10.1016/j.jcmg.2017.11.034 29454776 10.1016/j.jcmg.2017.11.034

[10] Hor KN, Gottliebson WM, Carson C, Wash E, Cnota J, Fleck R, et al Comparison of magnetic resonance feature tracking for strain calculation with harmonic phase imaging analysis. JACC Cardiovasc Imaging. 2010;3(2):144–51. doi: 10.1016/j.jcmg.2009.11.006 . 20159640 10.1016/j.jcmg.2009.11.006

[11] Schuster A, Hor KN, Kowallick JT, Beerbaum P, Kutty S. Cardiovascular magnetic resonance myocardial feature tracking. Circulation: Cardiovascular Imaging. 2016;9(4):e004077. 27009468 10.1161/CIRCIMAGING.115.004077

[12] Schuster A, Kutty S, Padiyath A, Parish V, Gribben P, Danford DA, et al Cardiovascular magnetic resonance myocardial feature tracking detects quantitative wall motion during dobutamine stress. Journal of Cardiovascular Magnetic Resonance. 2011;13(1):58. 21992220 10.1186/1532-429X-13-58 PMC3217847

[13] Morton G, Schuster A, Jogiya R, Kutty S, Beerbaum P, Nagel E. Inter-study reproducibility of cardiovascular magnetic resonance myocardial feature tracking. J Cardiovasc Magn Reson. 2012;14:43 Epub 2012/06/23. doi: 10.1186/1532-429X-14-43 ; PubMed Central PMCID: PMCPMC3461471. 22721175 10.1186/1532-429X-14-43 PMC3461471

[14] Schuster A, Morton G, Hussain ST, Jogiya R, Kutty S, Asrress KN, et al The intra-observer reproducibility of cardiovascular magnetic resonance myocardial feature tracking strain assessment is independent of field strength. Eur J Radiol. 2013;82(2):296–301. Epub 2012/12/19. doi: 10.1016/j.ejrad.2012.11.012 . 23246014 10.1016/j.ejrad.2012.11.012

[15] Orwat S, Kempny A, Diller GP, Bauerschmitz P, Bunck A, Maintz D, et al Cardiac magnetic resonance feature tracking: a novel method to assess myocardial strain. Comparison with echocardiographic speckle tracking in healthy volunteers and in patients with left ventricular hypertrophy. Kardiologia polska. 2014;72(4):363–71. Epub 2013/12/03. doi: 10.5603/KP.a2013.0319 . 24293146 10.5603/KP.a2013.0319

[16] Schuster A, Paul M, Bettencourt N, Morton G, Chiribiri A, Ishida M, et al Cardiovascular magnetic resonance myocardial feature tracking for quantitative viability assessment in ischemic cardiomyopathy. Int J Cardiol. 2013;166(2):413–20. doi: 10.1016/j.ijcard.2011.10.137 . 22130224 10.1016/j.ijcard.2011.10.137

[17] Heermann P, Hedderich DM, Paul M, Schulke C, Kroeger JR, Baessler B, et al Biventricular myocardial strain analysis in patients with arrhythmogenic right ventricular cardiomyopathy (ARVC) using cardiovascular magnetic resonance feature tracking. J Cardiovasc Magn Reson. 2014;16:75 doi: 10.1186/s12968-014-0075-z ; PubMed Central PMCID: PMCPMC4189682. 25315082 10.1186/s12968-014-0075-z PMC4189682

[18] Almeida-Morais L, Pereira-da-Silva T, Branco L, Timoteo AT, Agapito A, de Sousa L, et al The value of right ventricular longitudinal strain in the evaluation of adult patients with repaired tetralogy of Fallot: a new tool for a contemporary challenge. Cardiol Young. 2017;27(3):498–506. Epub 2016/05/27. doi: 10.1017/S1047951116000810 . 27226193 10.1017/S1047951116000810

[19] de Siqueira MEM, Pozo E, Fernandes VR, Sengupta PP, Modesto K, Gupta SS, et al Characterization and clinical significance of right ventricular mechanics in pulmonary hypertension evaluated with cardiovascular magnetic resonance feature tracking. Journal of Cardiovascular Magnetic Resonance. 2016;18:39 doi: 10.1186/s12968-016-0258-x 27306901 10.1186/s12968-016-0258-x PMC4910232

[20] Musa TA, Uddin A, Swoboda PP, Fairbairn TA, Dobson LE, Singh A, et al Cardiovascular magnetic resonance evaluation of symptomatic severe aortic stenosis: association of circumferential myocardial strain and mortality. Journal of Cardiovascular Magnetic Resonance. 2017;19:13 doi: 10.1186/s12968-017-0329-7 28173819 10.1186/s12968-017-0329-7 PMC5297161

[21] Schuster A, Stahnke VC, Unterberg-Buchwald C, Kowallick JT, Lamata P, Steinmetz M, et al Cardiovascular magnetic resonance feature-tracking assessment of myocardial mechanics: Intervendor agreement and considerations regarding reproducibility. Clin Radiol. 2015;70(9):989–98. Epub 2015/07/04. doi: 10.1016/j.crad.2015.05.006 ; PubMed Central PMCID: PMCPMC4683162. 26139384 10.1016/j.crad.2015.05.006 PMC4683162

[22] Bland JM, Altman DG. Statistical methods for assessing agreement between two methods of clinical measurement. Lancet. 1986;1(8476):307–10. Epub 1986/02/08. . 2868172

[23] Oppo K, Leen E, Angerson WJ, Cooke TG, McArdle CS. Doppler perfusion index: an interobserver and intraobserver reproducibility study. Radiology. 1998;208(2):453–7. doi: 10.1148/radiology.208.2.9680575 9680575 10.1148/radiology.208.2.9680575

[24] Grothues F, Smith GC, Moon JC, Bellenger NG, Collins P, Klein HU, et al Comparison of interstudy reproducibility of cardiovascular magnetic resonance with two-dimensional echocardiography in normal subjects and in patients with heart failure or left ventricular hypertrophy. Am J Cardiol. 2002;90(1):29–34. Epub 2002/06/29. . 12088775 10.1016/s0002-9149(02)02381-0

[25] Oztuna D EA, Tuccar E. Investigation of four different normality tests in terms of type 1 error rate and power under different distributions. Turkish Journal of Medical Sciences. 2006;36(3):171–6.

[26] Kowallick JT, Morton G, Lamata P, Jogiya R, Kutty S, Hasenfuß G, et al Quantification of atrial dynamics using cardiovascular magnetic resonance: inter-study reproducibility. J Cardiovasc Magn Reson. 2015;17(1). doi: 10.1186/s12968-015-0140-2 ; PubMed Central PMCID: PMCPMC4434799. 25982348 10.1186/s12968-015-0140-2 PMC4434799

[27] Augustine D, Lewandowski AJ, Lazdam M, Rai A, Francis J, Myerson S, et al Global and regional left ventricular myocardial deformation measures by magnetic resonance feature tracking in healthy volunteers: comparison with tagging and relevance of gender. Journal of Cardiovascular Magnetic Resonance. 2013;15(1):8 doi: 10.1186/1532-429x-15-8 23331550 10.1186/1532-429X-15-8 PMC3621526

[28] Kowallick JT, Lamata P, Hussain ST, Kutty S, Steinmetz M, Sohns JM, et al Quantification of left ventricular torsion and diastolic recoil using cardiovascular magnetic resonance myocardial feature tracking. PLoS One. 2014;9(10):e109164 doi: 10.1371/journal.pone.0109164 ; PubMed Central PMCID: PMCPMC4186780. 25285656 10.1371/journal.pone.0109164 PMC4186780

[29] Heyde B, Bouchez S, Thieren S, Vandenheuvel M, Jasaityte R, Barbosa D, et al Elastic Image Registration to Quantify 3-D Regional Myocardial Deformation from Volumetric Ultrasound: Experimental Validation in an Animal Model. Ultrasound in Medicine and Biology. 39(9):1688–97. doi: 10.1016/j.ultrasmedbio.2013.02.463 23791543 10.1016/j.ultrasmedbio.2013.02.463

[30] Morais P, Heyde B, Barbosa D, Queirós S, Claus P, D’hooge J. Cardiac Motion and Deformation Estimation from Tagged MRI Sequences Using a Temporal Coherent Image Registration Framework. In: Ourselin S, Rueckert D, Smith N, editors. Functional Imaging and Modeling of the Heart: 7th International Conference, FIMH 2013, London, UK, June 20–22, 2013 Proceedings. Berlin, Heidelberg: Springer Berlin Heidelberg; 2013. p. 316–24.

[31] Morais P, Marchi A, Bogaert JA, Dresselaers T, Heyde B, D'Hooge J, et al Cardiovascular magnetic resonance myocardial feature tracking using a non-rigid, elastic image registration algorithm: assessment of variability in a real-life clinical setting. J Cardiovasc Magn Reson. 2017;19(1):24 Epub 2017/02/18. doi: 10.1186/s12968-017-0333-y ; PubMed Central PMCID: PMCPMC5314711. 28209163 10.1186/s12968-017-0333-y PMC5314711

[32] Claus P, Omar AM, Pedrizzetti G, Sengupta PP, Nagel E. Tissue Tracking Technology for Assessing Cardiac Mechanics: Principles, Normal Values, and Clinical Applications. JACC Cardiovasc Imaging. 2015;8(12):1444–60. doi: 10.1016/j.jcmg.2015.11.001 . 26699113 10.1016/j.jcmg.2015.11.001

[33] Cheng S, Larson MG, McCabe EL, Osypiuk E, Lehman BT, Stanchev P, et al Reproducibility of Speckle-Tracking Based Strain Measures of Left Ventricular Function in a Community-Based Study. Journal of the American Society of Echocardiography: official publication of the American Society of Echocardiography. 2013;26(11):1258–66. doi: 10.1016/j.echo.2013.07.002 23953701 10.1016/j.echo.2013.07.002 PMC3812381

[34] Voigt JU, Pedrizzetti G, Lysyansky P, Marwick TH, Houle H, Baumann R, et al Definitions for a common standard for 2D speckle tracking echocardiography: consensus document of the EACVI/ASE/Industry Task Force to standardize deformation imaging. Eur Heart J Cardiovasc Imaging. 2015;16(1):1–11. Epub 2014/12/20. doi: 10.1093/ehjci/jeu184 . 25525063 10.1093/ehjci/jeu184

[35] Donekal S, Ambale-Venkatesh B, Berkowitz S, Wu CO, Choi EY, Fernandes V, et al Inter-study reproducibility of cardiovascular magnetic resonance tagging. Journal of Cardiovascular Magnetic Resonance. 2013;15(1):37 doi: 10.1186/1532-429x-15-37 23663535 10.1186/1532-429X-15-37 PMC3667053

[36] Kang Y, Cheng L, Li L, Chen H, Sun M, Wei Z, et al Early detection of anthracycline-induced cardiotoxicity using two-dimensional speckle tracking echocardiography. Cardiology journal. 2013;20(6):592–9. Epub 2013/12/18. doi: 10.5603/CJ.2013.0158 . 24338535 10.5603/CJ.2013.0158

[37] Tadic M, Majstorovic A, Pencic B, Ivanovic B, Neskovic A, Badano L, et al The impact of high-normal blood pressure on left ventricular mechanics: a three-dimensional and speckle tracking echocardiography study. Int J Cardiovasc Imaging. 2014;30(4):699–711. Epub 2014/02/04. doi: 10.1007/s10554-014-0382-3 . 24487950 10.1007/s10554-014-0382-3

