# Correction: Inter-vendor reproducibility of left and right ventricular cardiovascular magnetic resonance myocardial feature-tracking

**DOI:** 10.1371/journal.pone.0199489

**Published:** 2018-06-18

**Authors:** Roman Johannes Gertz, Torben Lange, Johannes Tammo Kowallick, Sören Jan Backhaus, Michael Steinmetz, Wieland Staab, Shelby Kutty, Gerd Hasenfuß, Joachim Lotz, Andreas Schuster

The x-axis labels for panels g and h of Fig 2 do not appear. Please see the complete, correct Fig 2 here.

**Fig 2:**
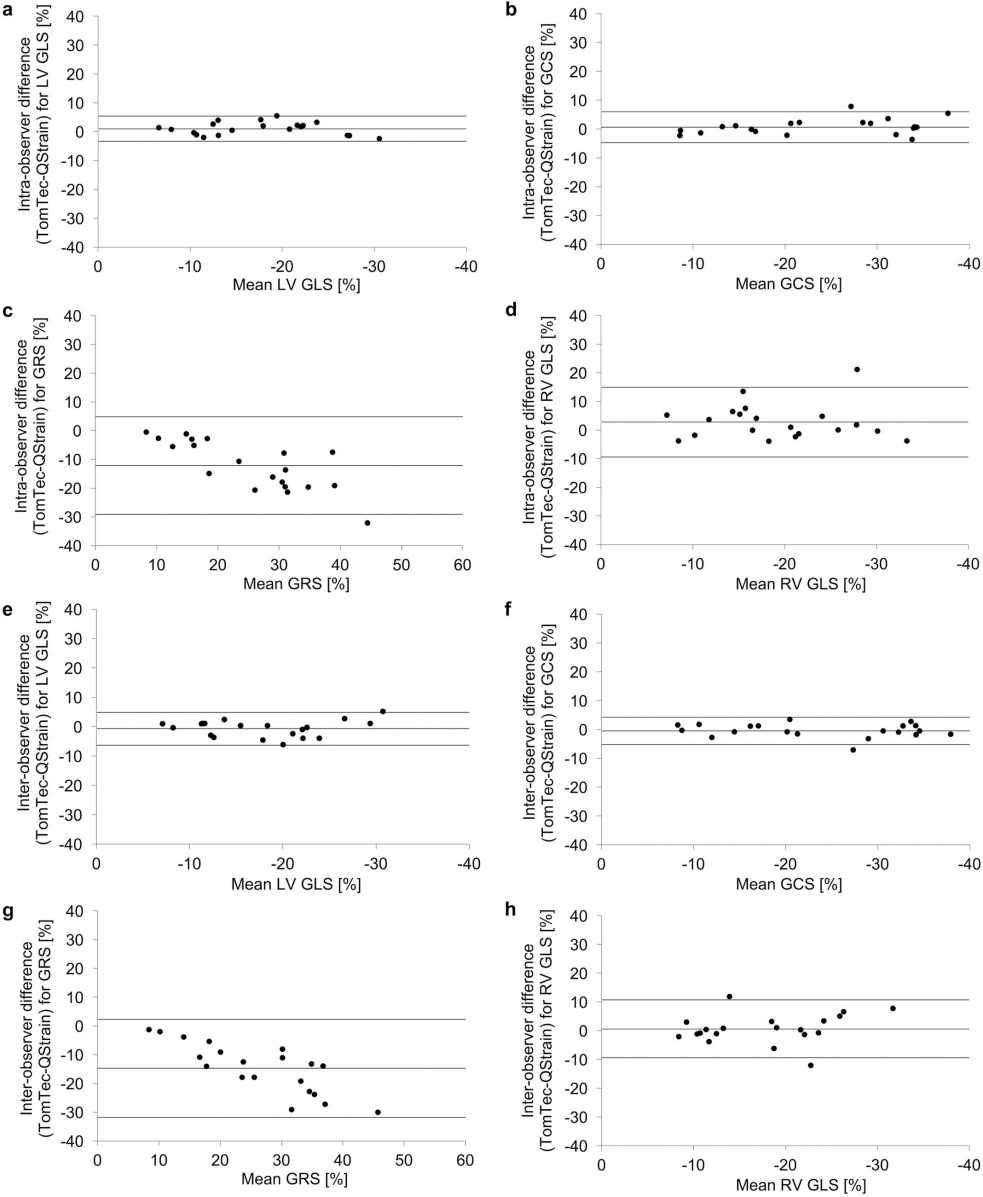
Reproducibility for CMR-FT derived global strain parameters at intra- and inter-observer levels. Inter-vendor agreement for global strain parameters for healthy volunteers and patients with impaired cardiac output based on three averaged measurements (R3). *Panel a–d*: Bland-Altman plots with limits of agreement (95% confidence intervals) demonstrating the CMR-FT derived reproducibility at an intra-observer level are being displayed. *Panel e–h*: Bland-Altman plots with limits of agreement (95% confidence intervals) demonstrating the CMR-FT derived reproducibility at an inter-observer level are being displayed.
