# Supplementary figures and images for: Inter-vendor reproducibility of left and right ventricular cardiovascular magnetic resonance myocardial feature-tracking
Source: PLoS One. 2018 Mar 14;13(3):e0193746. doi: 10.1371/journal.pone.0193746 (PMC5851552; doi:10.1371/journal.pone.0193746)

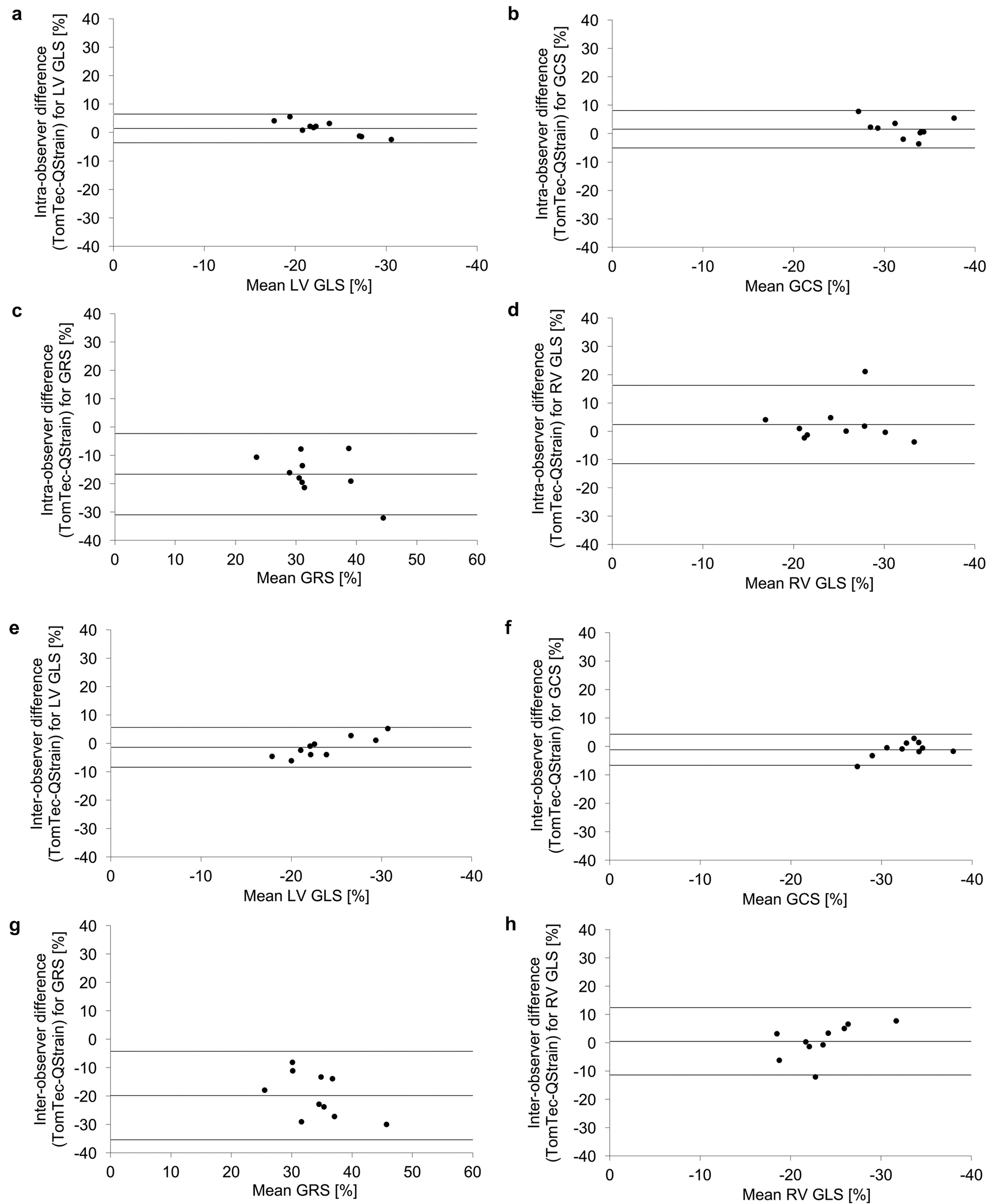

Supplement: S1 Fig — Inter-vendor agreement for global strain parameters for normal subjects based on three averaged measurements (R3). Panel a–d: Bland-Altman plots with limits of agreement (95% confidence intervals) demonstrating the CMR-FT derived reproducibility at an intra-observer level are being displayed. Panel e–h: Bland-Altman plots with limits of agreement (95% confidence intervals) demonstrating the CMR-FT derived reproducibility at an inter-observer level are being displayed. (TIF) [file pone.0193746.s001.tif]

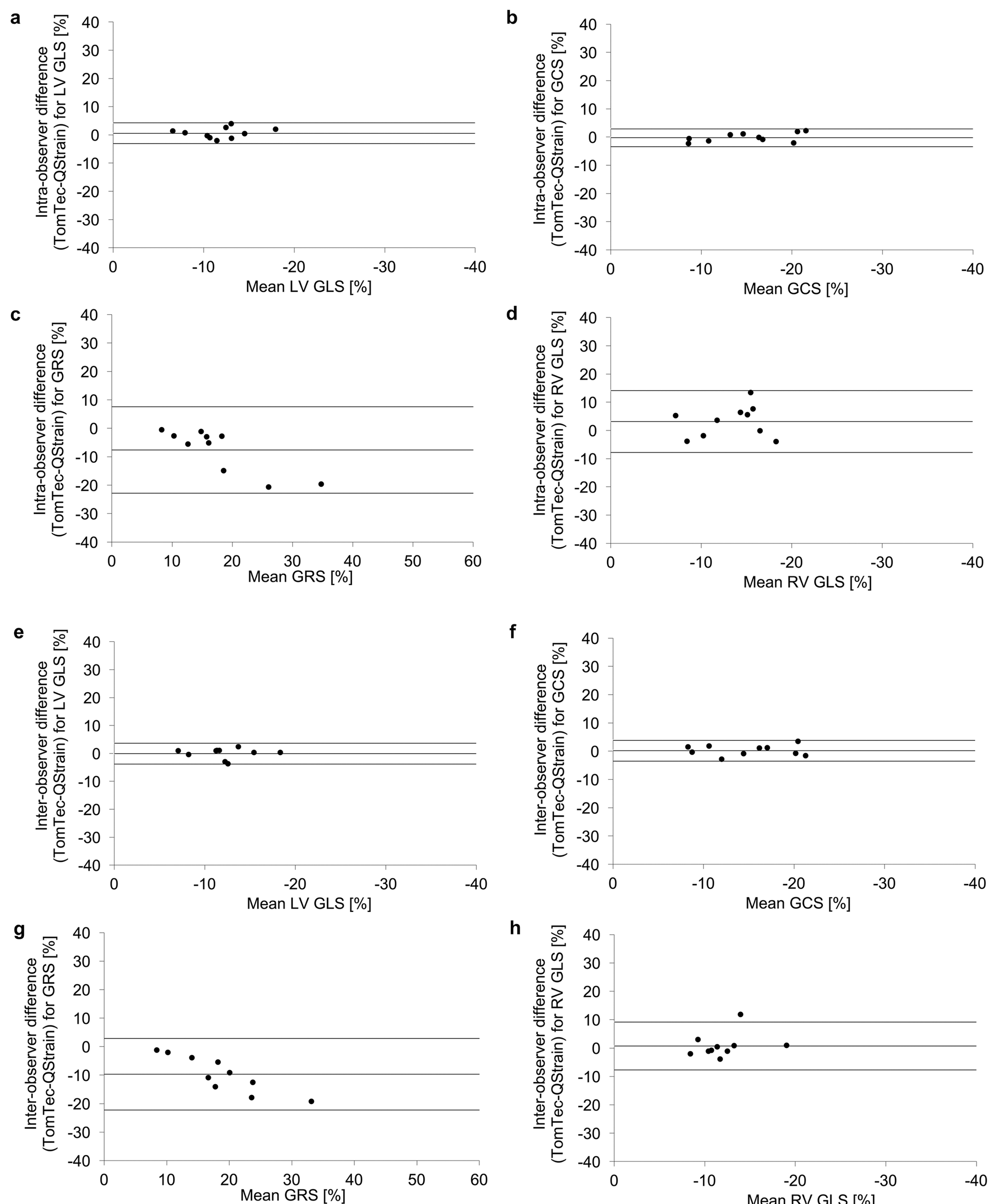

Supplement: S2 Fig — Inter-vendor agreement for global strain parameters for patients with impaired cardiac function as defined by reduced ejection fraction based on three averaged measurements (R3). Panel a–d: Bland-Altman plots with limits of agreement (95% confidence intervals) demonstrating the CMR-FT derived reproducibility at an intra-observer level are being displayed. Panel e–h: Bland-Altman plots with limits of agreement (95% confidence intervals) demonstrating the CMR-FT derived reproducibility at an inter-observer level are being displayed. (TIF) [file pone.0193746.s002.tif]
