# Supplementary material for: Inter-vendor reproducibility of left and right ventricular cardiovascular magnetic resonance myocardial feature-tracking
Source: PLoS One. 2018 Mar 14;13(3):e0193746. doi: 10.1371/journal.pone.0193746 (PMC5851552; doi:10.1371/journal.pone.0193746)
Supplement: S1 Table — SD, standard deviation; Diff., differences; ICC, intra-class correlation coefficient; CoV, coefficient of variation; CI, confidence interval; LV GLS, global left ventricular longitudinal strain; GCS, global left ventricular circumferential strain; GRS, global left ventricular radial strain; RV GLS, global right ventricular longitudinal strain. (DOCX) [file pone.0193746.s003.docx]

|  | | | | | | | | | | | |
| --- | --- | --- | --- | --- | --- | --- | --- | --- | --- | --- | --- |
|  |  | TomTec versus QStrain | |  | TomTec |  |  | QStrain |  |  |  |
|  |  | Mean Difference (SD of the Diff.) | ICC (95% CI) | CoV (%) | Mean Difference (SD of the Diff.) | ICC (95% CI) | CoV (%) | Mean Difference (SD of the Diff.) | ICC (95% CI) | CoV (%) |  |
|  |  |  |  |  |  |  |  |  |  |  |  |
| Intra-observer | LV GLS % | 1.40 (2.56) | 0.88 (0.52–0.97) | 10.99 | -0.17 (0.58) | 1.00 (0.99–1.00) | 2.58 | 0.39 (1.11) | 0.96 (0.84–0.99) | 4.59 |  |
|  | GCS % | 1.57 (3.36) | 0.70 (0.00–0.92) | 10.41 | -0.52 (0.75) | 0.99 (0.94–1.00) | 2.42 | 0.23 (0.92) | 0.97 (0.90–0.99) | 2.78 |  |
|  | GRS % | -16.66 (7.32) | 0.22 (0.00–0.68) | 22.18 | -1.37 (2.92) | 0.93 (0.73–0.98) | 11.51 | -2.84 (4.36) | 0.90 (0.58–0.98) | 10.21 |  |
|  | RV GLS % | 2.42 (7.07) | 0.49 (0.00–0.87) | 28.32 | -1.41 (0.42) | 0.98 (0.09–1.00) | 1.83 | 0.72 (1.51) | 0.98 (0.94–1.00) | 5.71 |  |
|  |  |  |  |  |  |  |  |  |  |  |  |
| Inter-observer | LV GLS % | -1.40 (3.58) | 0.80 (0.27–0.95) | 15.12 | 0.39 (1.08) | 0.99 (0.96–1.00) | 4.76 | 0.22 (0.61) | 0.99 (0.96–1.00) | 2.54 |  |
|  | GCS % | -1.18 (2.77) | 0.78 (0.20–0.94) | 8.48 | 0.62 (1.09) | 0.98 (0.90–0.99) | 3.43 | 0.30 (0.90) | 0.98 (0.92–0.99) | 2.71 |  |
|  | GRS % | -19.84 (7.92) | 0.11 (0.00–0.51) | 23.13 | 0.34 (3.48) | 0.87 (0.47–0.97) | 14.20 | 6.89 (6.74) | 0.68 (0.00–0.92) | 17.79 |  |
|  | RV GLS % | 0.51 (6.07) | 0.41 (0.00–0.86) | 25.76 | 0.09 (0.71) | 1.00 (0.99–1.00) | 2.98 | 0.10 (1.00) | 0.99 (0.98–1.00) | 3.82 |  |
|  | | | | | | | | | | | |
