# Supplementary material for: Inter-vendor reproducibility of left and right ventricular cardiovascular magnetic resonance myocardial feature-tracking
Source: PLoS One. 2018 Mar 14;13(3):e0193746. doi: 10.1371/journal.pone.0193746 (PMC5851552; doi:10.1371/journal.pone.0193746)
Supplement: S2 Table — SD, standard deviation; Diff., differences; ICC, intra-class correlation coefficient; CoV, coefficient of variation; CI, confidence interval; LV GLS, global left ventricular longitudinal strain; GCS, global left ventricular circumferential strain; GRS, global left ventricular radial strain; RV GLS, global right ventricular longitudinal strain; *as defined by reduced ejection fraction. (DOCX) [file pone.0193746.s004.docx]

|  | | | | | | | | | | |
| --- | --- | --- | --- | --- | --- | --- | --- | --- | --- | --- |
|  |  | TomTec versus QStrain | |  | TomTec |  |  | QStrain |  |  |
|  |  | Mean Difference (SD of the Diff.) | ICC (95% CI) | CoV (%) | Mean Difference (SD of the Diff.) | ICC (95% CI) | CoV (%) | Mean Difference (SD of the Diff.) | ICC (95% CI) | CoV (%) |
|  |  |  |  |  |  |  |  |  |  |  |
| Intra-observer | LV GLS % | 0.60 (1.89) | 0.91 (0.67–0.98) | 15.99 | -0.13 (0.73) | 0.99 (0.95–1.00) | 6.38 | 0.10 (0.56) | 0.99 (0.98–1.00) | 4.62 |
|  | GCS % | -0.26 (1.61) | 0.97 (0.90–0.99) | 10.60 | -0.45 (0.29) | 1.00 (0.84–1.00) | 1.93 | -0.17 (0.47) | 1.00 (0.99–1.00) | 3.13 |
|  | GRS % | -7.65 (7.75) | 0.62 (0.00–0.90) | 44.05 | -0.34 (3.16) | 0.89 (0.55–0.97) | 22.63 | -2.07 (5.01) | 0.94 (0.77–0.98) | 22.30 |
|  | RV GLS % | 3.13 (5.59) | 0.38 (0.00–0.83) | 42.00 | 0.17 (1.09) | 0.98 (0.93–1.00) | 9.23 | 0.46 (1.29) | 0.98 (0.94–1.00) | 8.54 |
|  |  |  |  |  |  |  |  |  |  |  |
| Inter-observer | LV GLS % | -0.05 (1.91) | 0.92 (0.67–0.98) | 15.61 | 0.64 (0.80) | 0.98 (0.85–1.00) | 6.75 | 0.14 (0.84) | 0.99 (0.95–1.00) | 6.91 |
|  | GCS % | 0.15 (1.87) | 0.97 (0.87–0.99) | 12.54 | -0.28 (0.93) | 0.99 (0.96–1.00) | 6.13 | 0.16 (0.96) | 0.99 (0.97–1.00) | 6.33 |
|  | GRS % | -9.73 (6.40) | 0.56 (0.00–0.89) | 34.36 | 0.01 (2.61) | 0.93 (0.70–0.98) | 18.97 | 2.13 (6.45) | 0.88 (0.55–0.97) | 31.68 |
|  | RV GLS % | 0.74 (4.31) | 0.50 (0.00–0.88) | 35.64 | 0.71 (1.41) | 0.97 (0.87–0.99) | 11.63 | 0.28 (0.59) | 1.00 (0.99–1.00) | 3.90 |
|  | | | | | | | | | | |
